# Supplementary material for: VO2 memristor-based frequency converter with in-situ synthesize and mix for wireless internet-of-things
Source: Nat Commun. 2024 Feb 19;15:1523. doi: 10.1038/s41467-024-45923-7 (PMC10876666; doi:10.1038/s41467-024-45923-7)
Supplement: Supplementary file 1 — Supplementary Information [file 41467_2024_45923_MOESM1_ESM.pdf]

**Supplementary Information**

# $VO_2$ memristor-based frequency converter enabling in-situ synthesize and mix for wireless internet-of-things

*Chang Liu*[1], *Pek Jun Tiw*[1], *Teng Zhang*[1], *Yanghao Wang*[1], *Lei Cai*[1], *Rui Yuan*[1], *Zelun Pan*[1], *Wenshuo Yue*[1], *Yaoyu Tao*[1,2*], *and Yuchao Yang*[1,2,3,4*]

[1] Beijing Advanced Innovation Center for Integrated Circuits, School of Integrated Circuits, Peking University, Beijing 100871, China

[2] Center for Brain Inspired Chips, Institute for Artificial Intelligence, Frontiers Science Center for Nano-optoelectronics, Peking University, Beijing 100871, China

[3] School of Electronic and Computer Engineering, Peking University, Shenzhen 518055, China

[4] Center for Brain Inspired Intelligence, Chinese Institute for Brain Research (CIBR), Beijing, Beijing 102206, China

E-mail: taoyaoyutyy@pku.edu.cn, yuchaoyang@pku.edu.cn

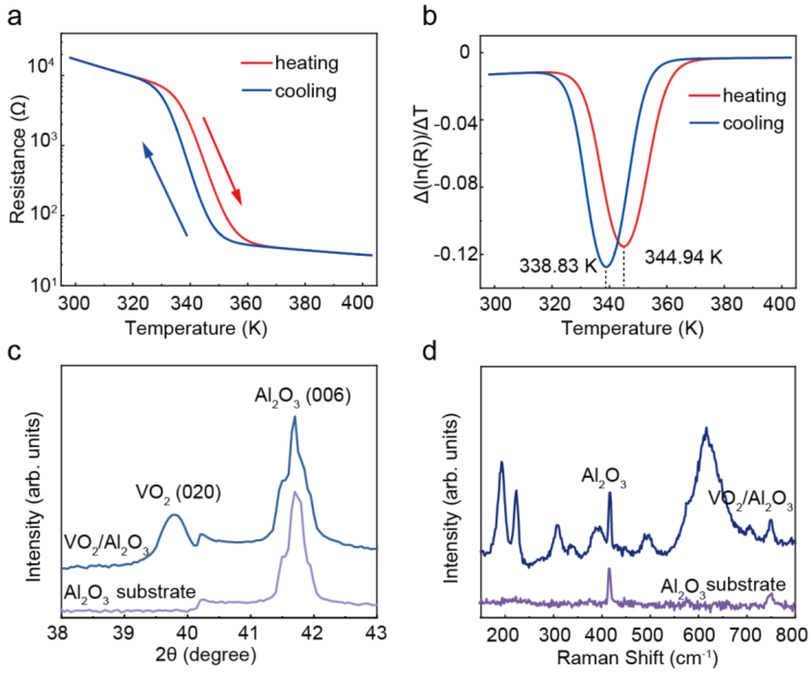


**Supplementary Figure 1. Characterization of $VO_2$ memristor. (a)** Temperature-dependent resistance switching plot and the corresponding **(b)** differential curve of the $VO_2$ film. The heating and cooling branches are represented by the red and blue curves, respectively. **(c)** Comparison of XRD pattern before and after $VO_2$ film deposition. **(d)** Comparison of Raman spectra before and after $VO_2$ film deposition.

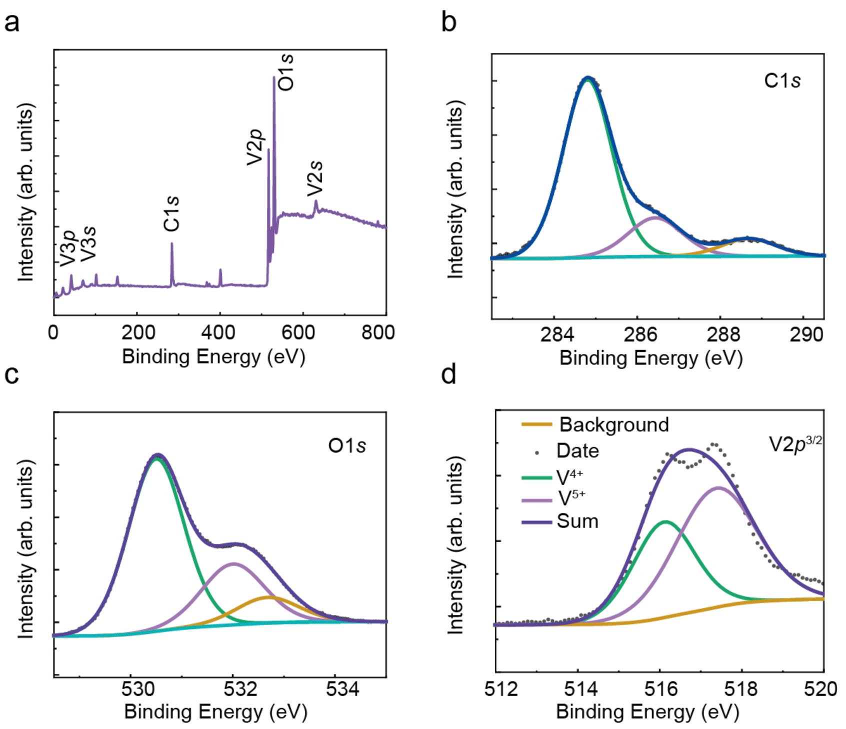


**Supplementary Figure 2. X-ray photoelectron spectrum (XPS) analysis.** (a) XPS spectra survey and high-resolution (b) C 1*s*, (c) O 1*s* and (d) V 2*p* core level spectra for $VO_2$ film.

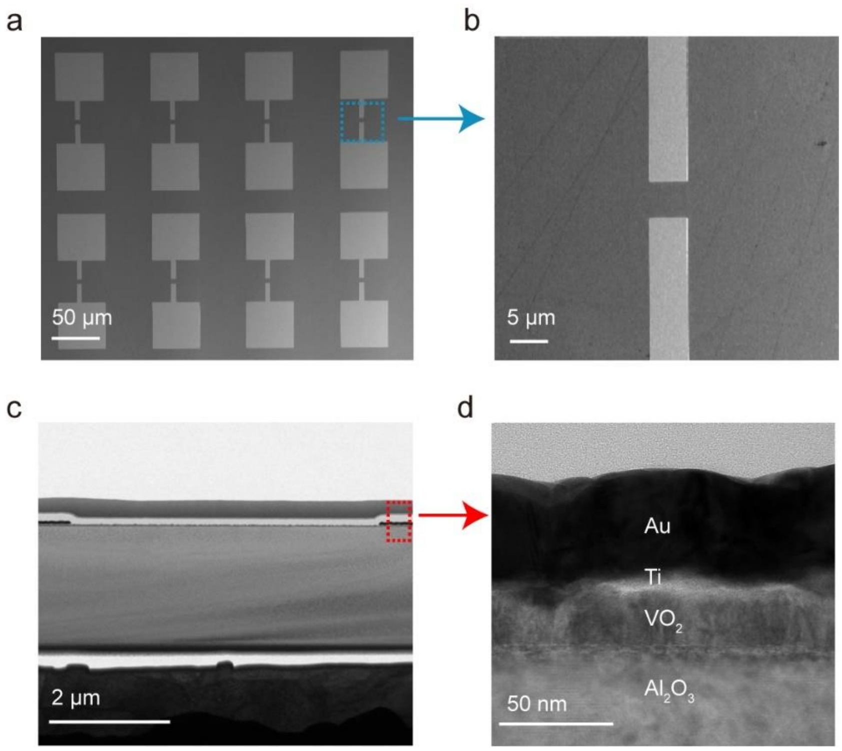


**Supplementary Figure 3. Microstructure of $VO_2$ memristor. (a)** SEM image of the epitaxial $VO_2$ device. **(b)** Zoom-in views of the channel regions in SEM. **(c)** Cross-sectional transmission electron microscopy (TEM) image of the epitaxial $VO_2$ device. (d) A closer view of the $VO_2$ device in TEM.

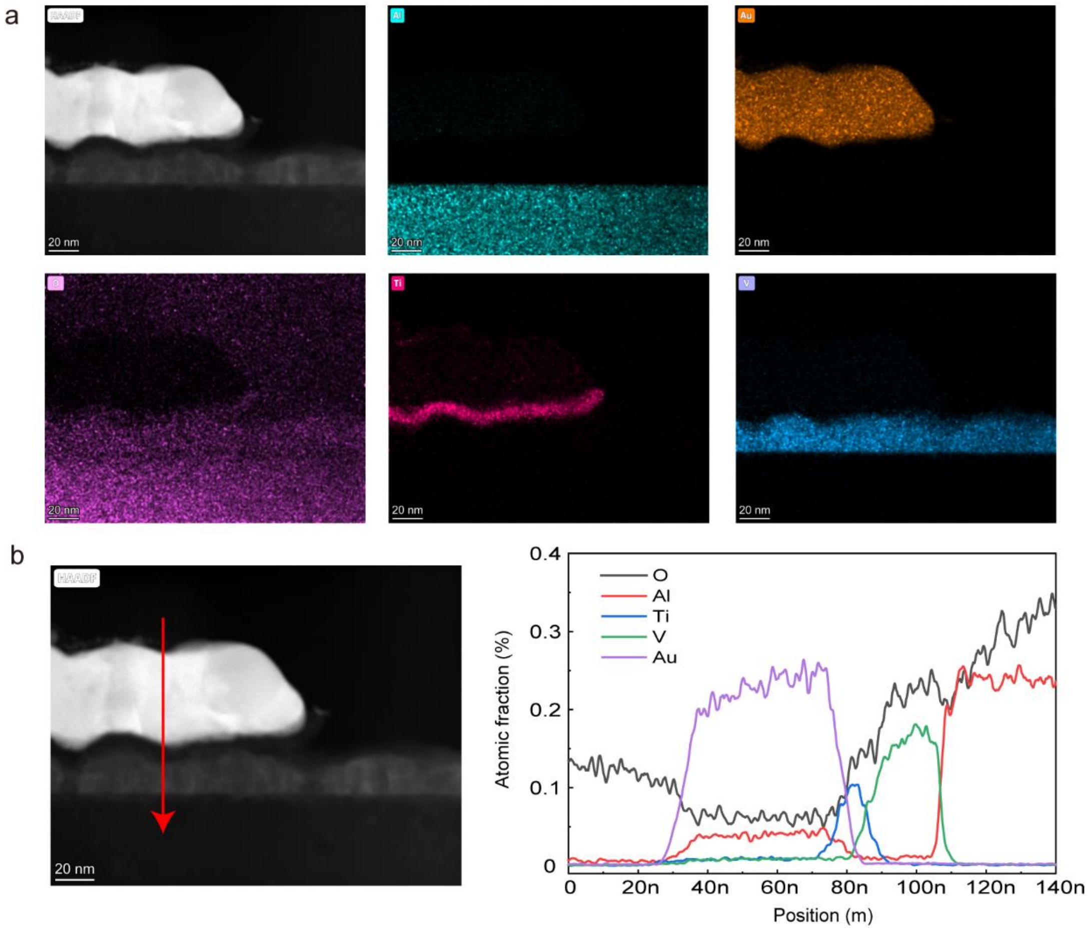


**Supplementary Figure 4. The compositional characterization of $VO_2$ memristor. (a)** Cross-sectional STEM image and the elemental mapping of Al, Au, O, Ti and V in the device. **(b)** EDS elemental line-scan in the location of the $VO_2$ device shown by the STEM image.

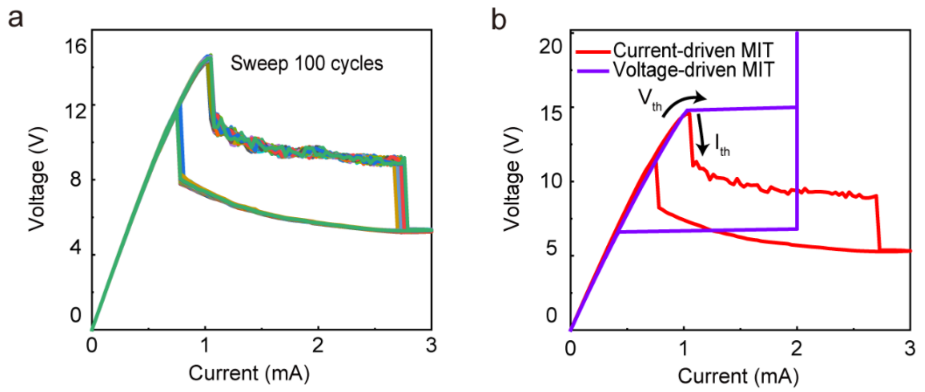


**Supplementary Figure 5. Voltage- and current-driven metal-insulator transition (MIT) in $VO_2$ memristor. (a)** Quasi-static *I-V* characteristics of the device repeated for 100 cycles. **(b)** Current–voltage response of voltage- (red) and current-driven (purple) MITs in $VO_2$ device. The threshold switching voltage ($V_{th}$) and threshold switching current ($I_{th}$) are labeled. While the voltage-driven MIT occurs abruptly, the current-driven MIT occurs over a range of current values.

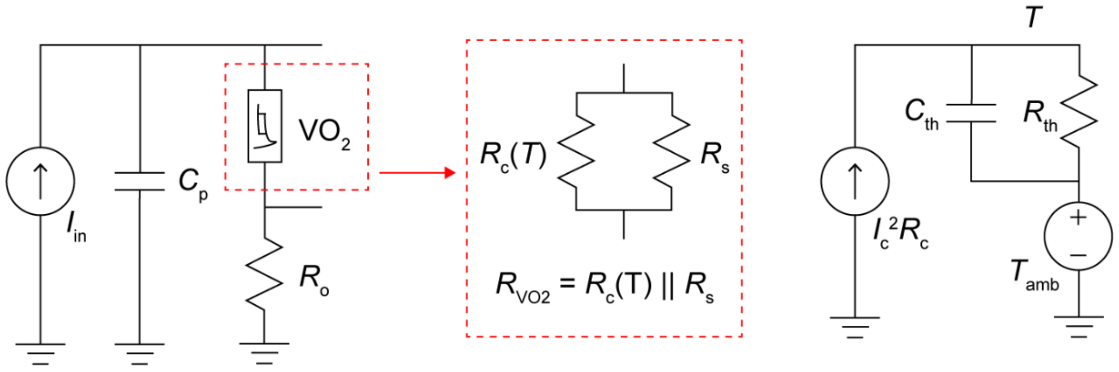


**Supplementary Figure 6. The $VO_2$ memristor SPICE model.** The current-driven circuit on the left is the setup used to measure the *I*-*V* characteristics and the oscillatory behavior of the memristor. $C_p$ includes the parasitic capacitance of the measurement circuit and externally connected capacitors, if applicable. $R_o$ is a readout resistor. The memristor is modelled as two resistors in parallel, where $R_s$ is fixed while $R_c$ is dependent on the internal temperature of the device. The time evolution of this temperature is given by the thermal circuit on the right, which implements Newton's law of cooling. Joule heating and the ambient temperature is modelled by a current source ($I_c^2R_c$) and a voltage source ($T_{amb}$), respectively. $R_{th}$ and $C_{th}$ represents the thermal resistance and capacitance, respectively.

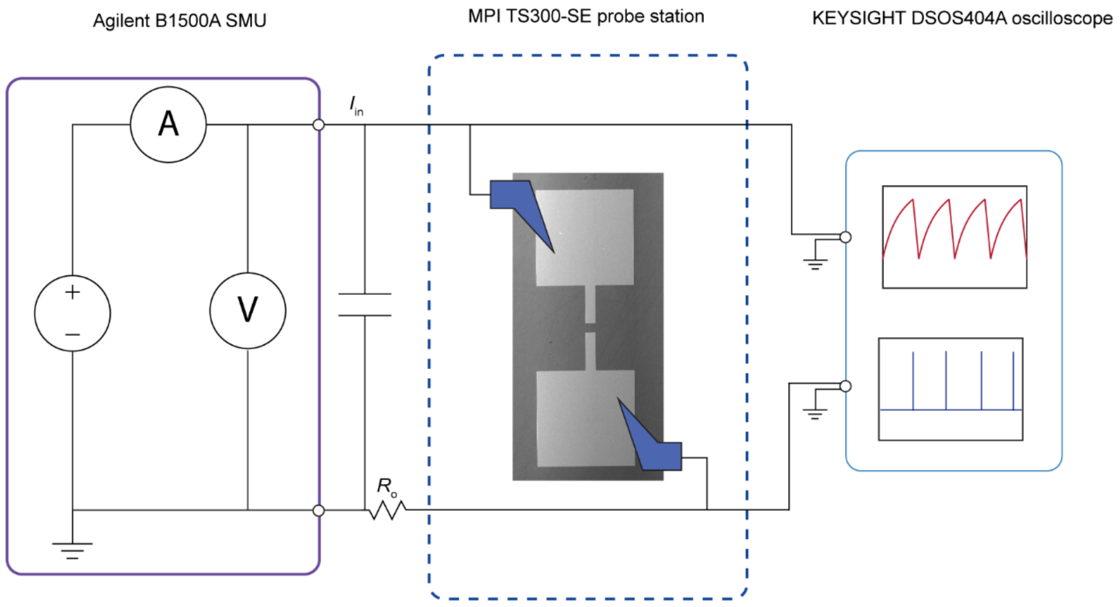


**Supplementary Figure 7. Experimental setup to measure the behavior of the oscillator circuit.** The $VO_2$ device is placed in a MPI TS300-SE probe station for easy connection to external circuit, which includes an Agilent B1500 source measure unit and a KEYSIGHT DSOS404A oscilloscope.

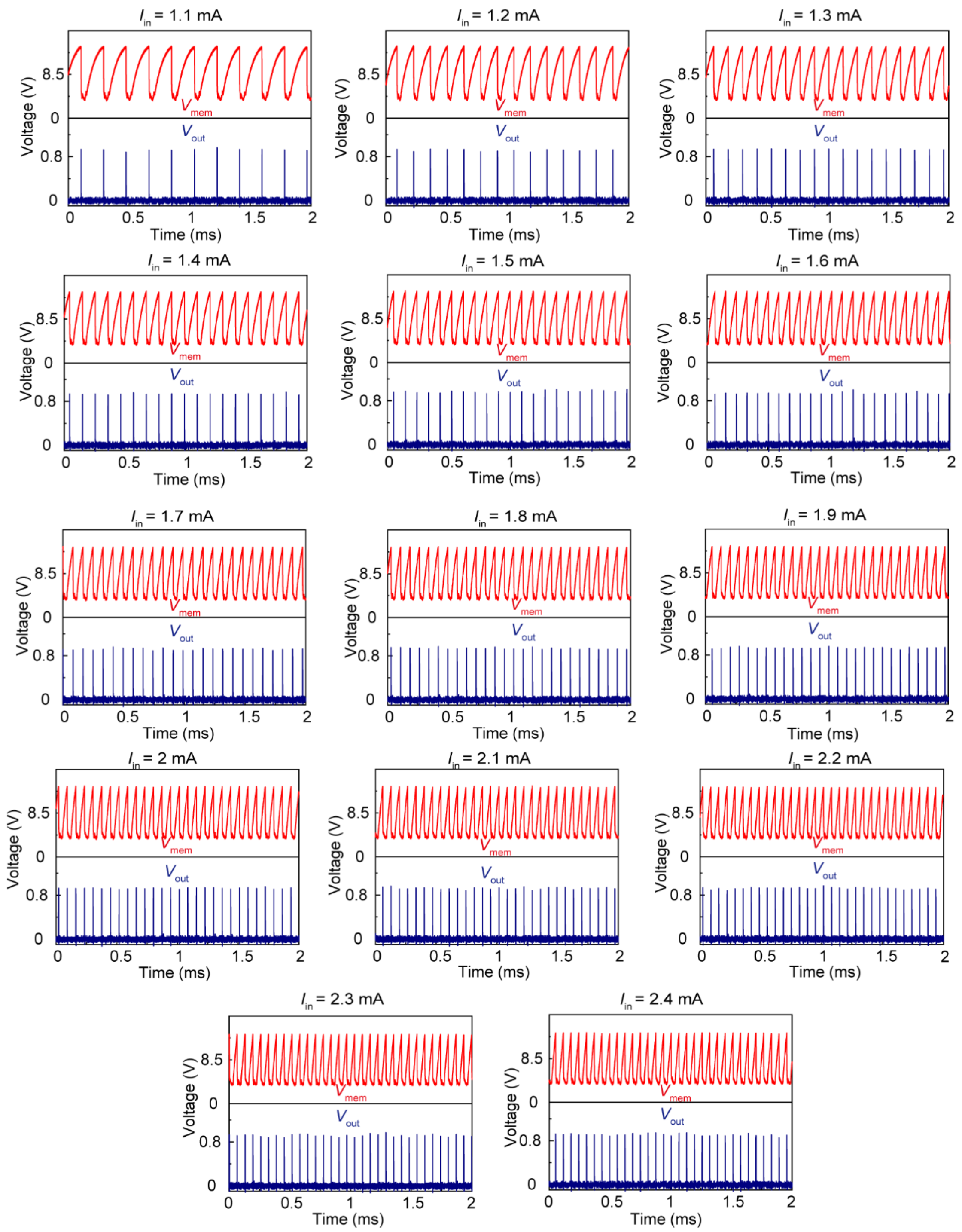


**Supplementary Figure 8. Output results of the oscillator using different applied currents.** The figure shows additional data for different series applied currents $I_{\text{in}}$. The higher the current, the faster the charge, resulting in an increase of the spiking frequency.

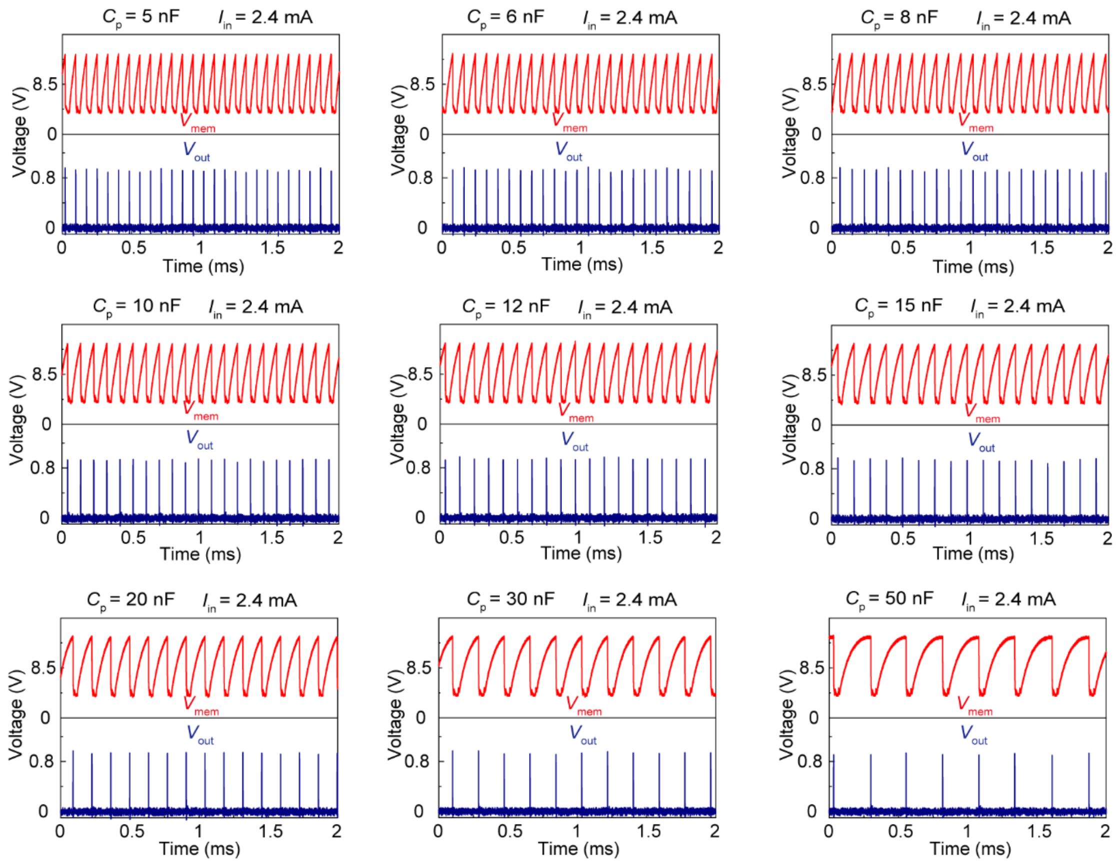


**Supplementary Figure 9. Output results of the oscillator using different parallel capacitors.** The figure shows additional data for different series parallel capacitors.

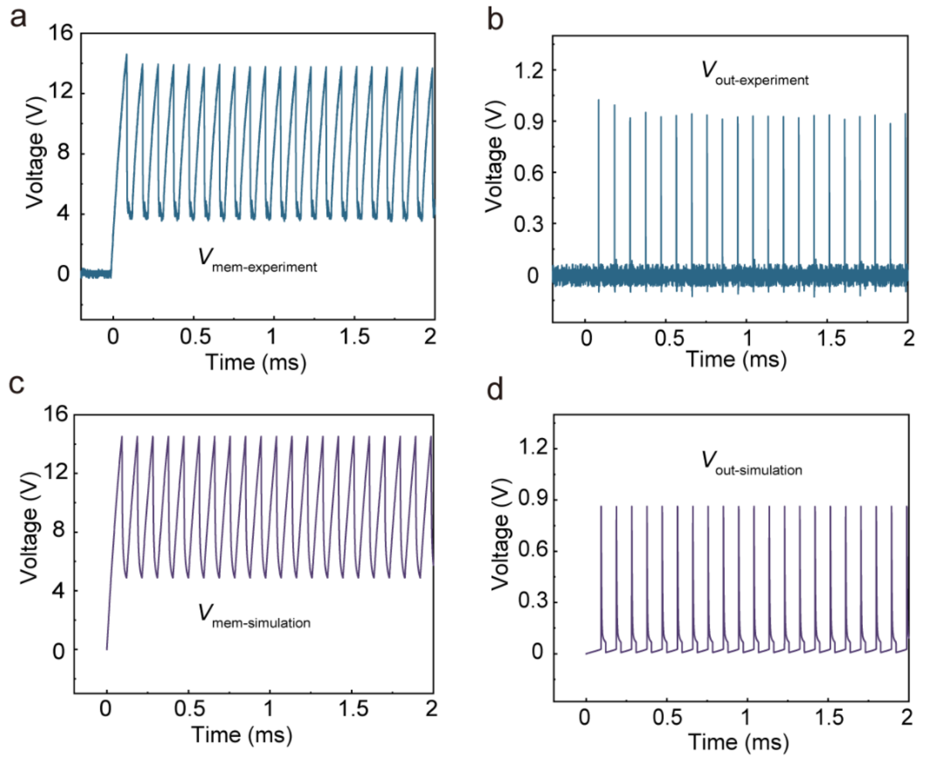


**Supplementary Figure 10. Experiment and simulation results of oscillator under 2.4 mA applied current. (a-b)** Experiment results of oscillator under 2.4 mA applied current with a 10 nF parallel capacitor. **(c-d)** Simulation results of oscillator under 2.4 mA applied current with a 12.05 nF parallel capacitor. The simulation and experiment results are in good agreement.

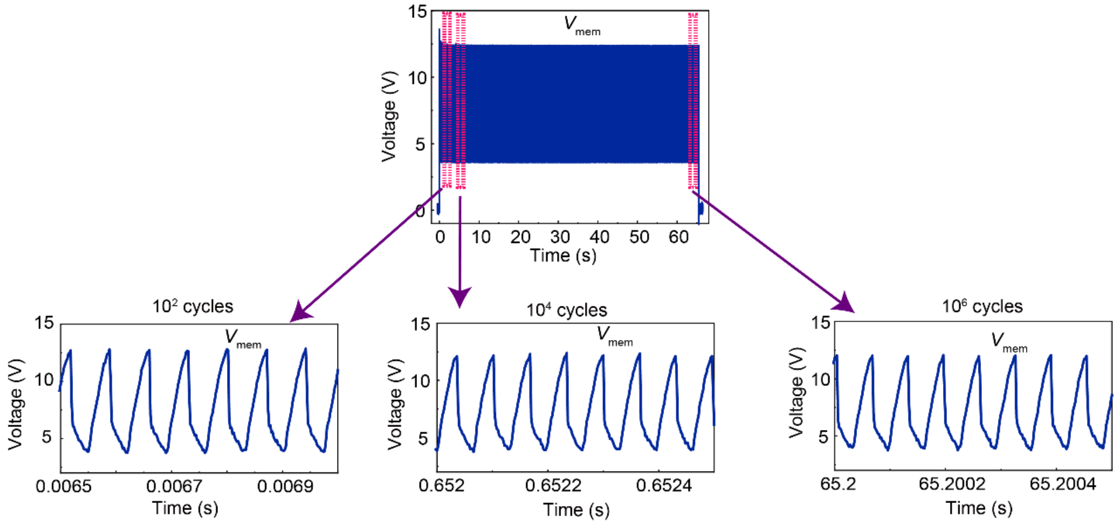


**Supplementary Figure 11. Endurance of the $VO_2$ oscillator.** Experiment results of oscillator under 2.1 mA applied current with a width of 66 s.

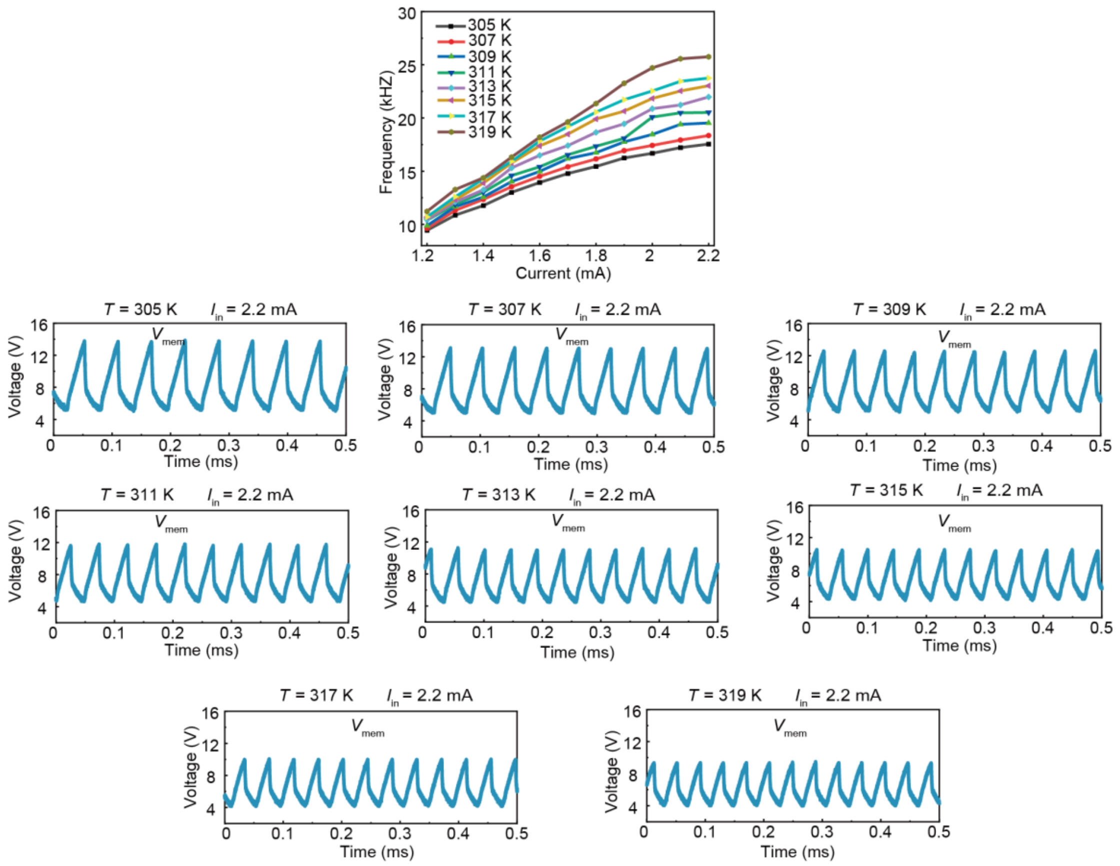


**Supplementary Figure 12. Oscillation frequency response under different temperature.** As the temperature increases, the oscillation frequency increases.

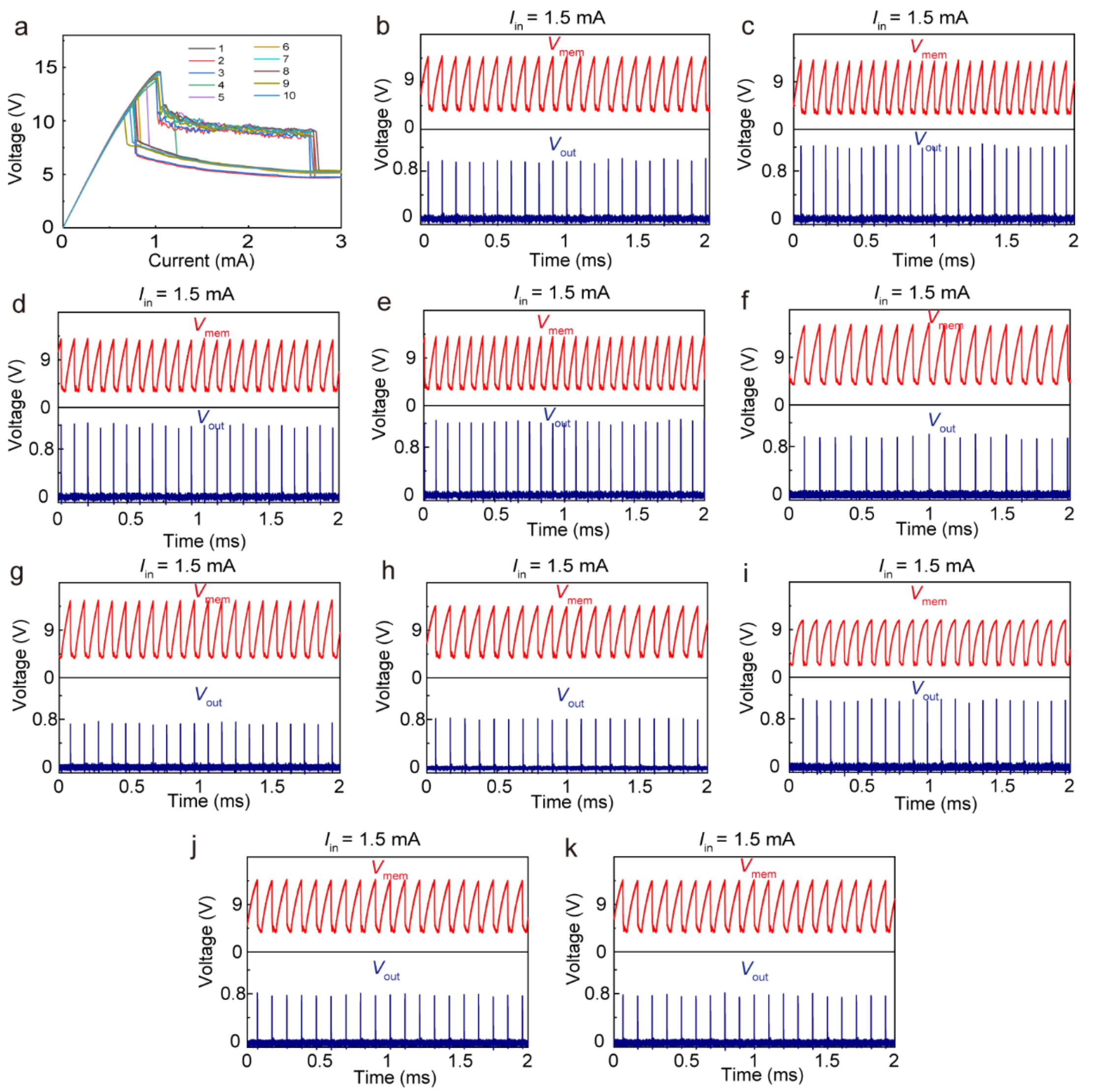


**Supplementary Figure 13. Device to device and oscillator to oscillator variations of the $VO_2$ memristor. (a)** Quasi-static *I-V* characteristics of the $VO_2$ device measured in 10 different devices. **(b-k)** Experiment results of oscillators under 1.5 mA applied current with 10 different $VO_2$ devices.

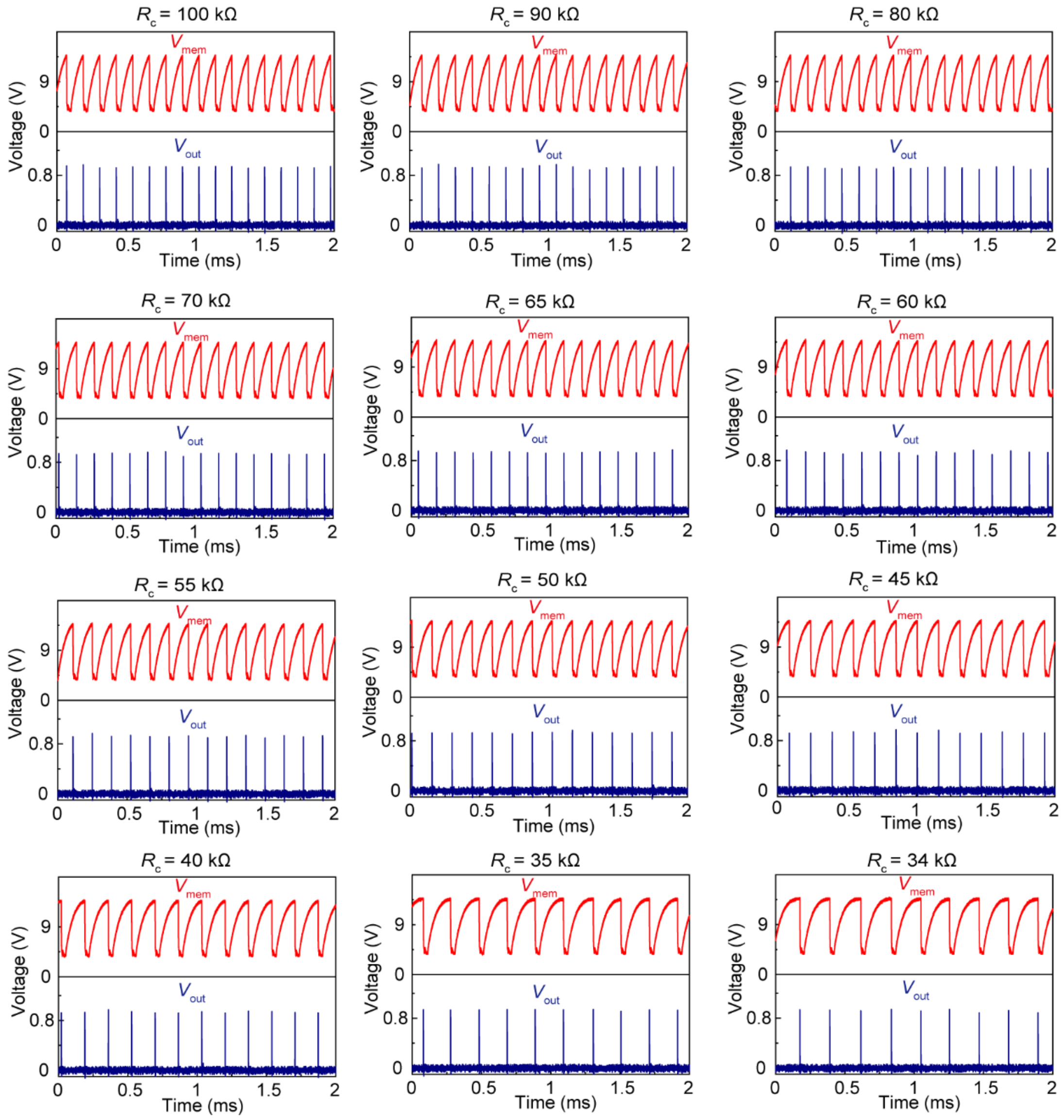


**Supplementary Figure 14. Output results of the oscillator using different calibration resistances $R_c$.** The figure shows additional data for different series calibration resistances $R_c$.

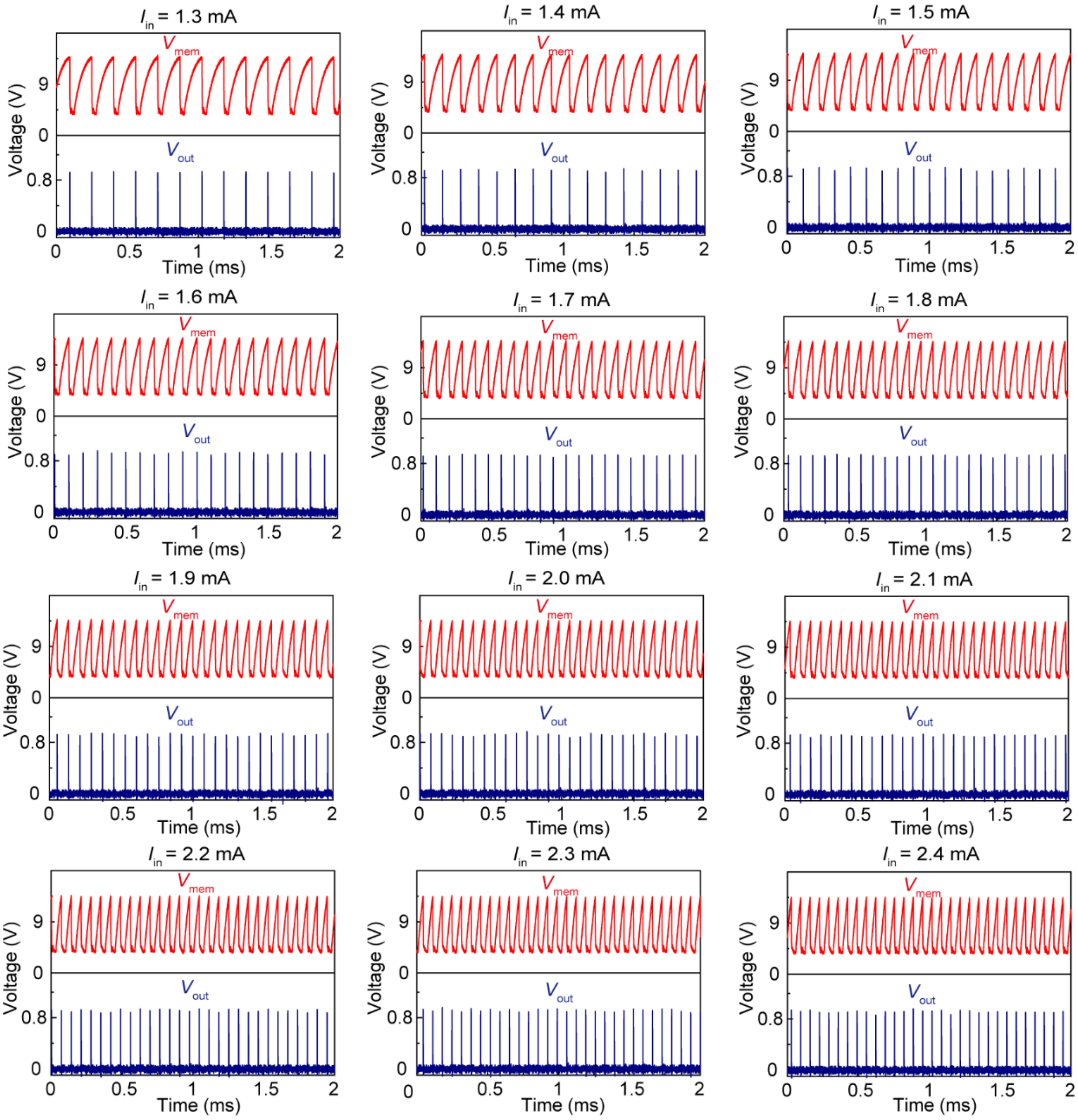


**Supplementary Figure 15. Output results of the oscillator using different applied currents under 70 kΩ calibration resistances.** The figure shows additional data for different series applied currents $I_{in}$.

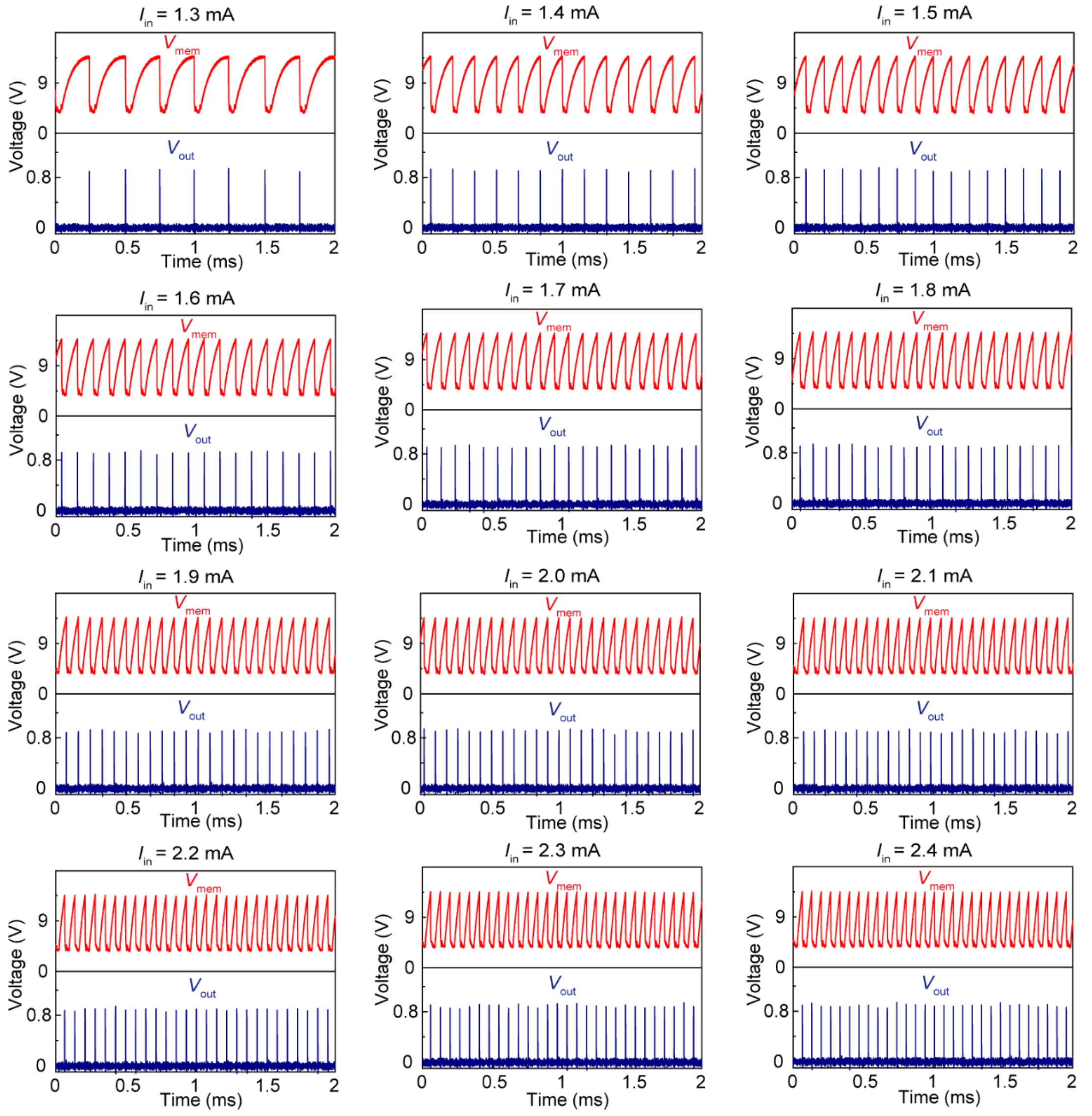


**Supplementary Figure 16. Output results of the oscillator using different applied currents under 45 kΩ calibration resistances.** The figure shows additional data for different series applied currents $I_{in}$.

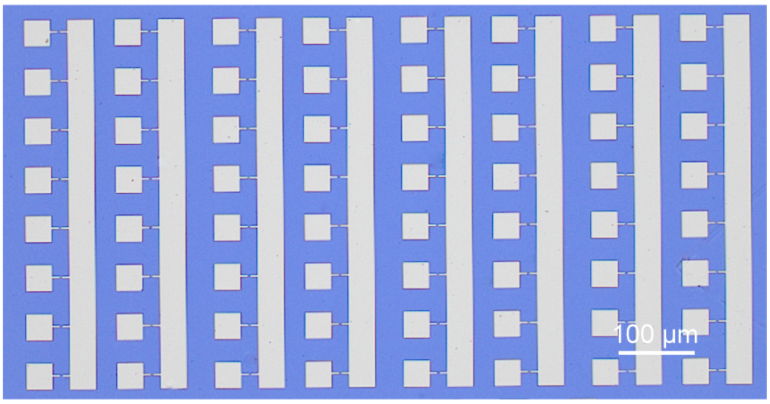


**Supplementary Figure 17. Optical image of 8×8 array of the $VO_2$ memristor.** The array consists of eight 8×1 arrays, the floating electrodes are used to connect the current source, and the other electrodes are interconnected.

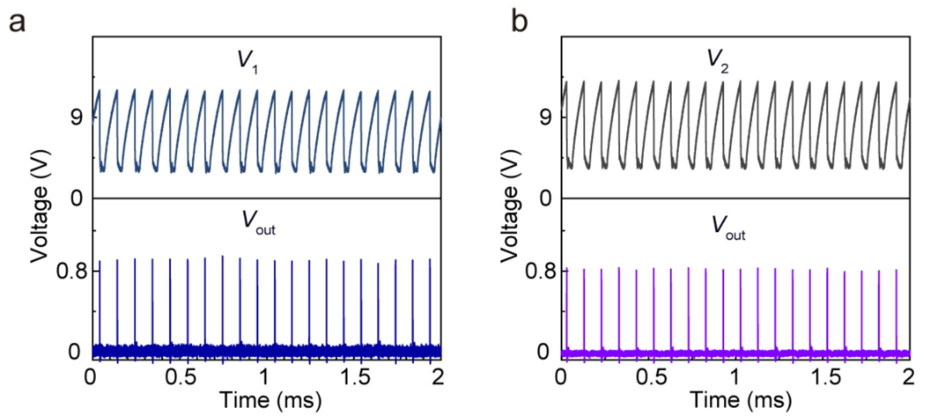


**Supplementary Figure 18. The output results of oscillator under different applied current. (a** and **b)** Experiment results of oscillator under only one current bias applied with 1.41 mA (a) and 1.42 mA (b), respectively.

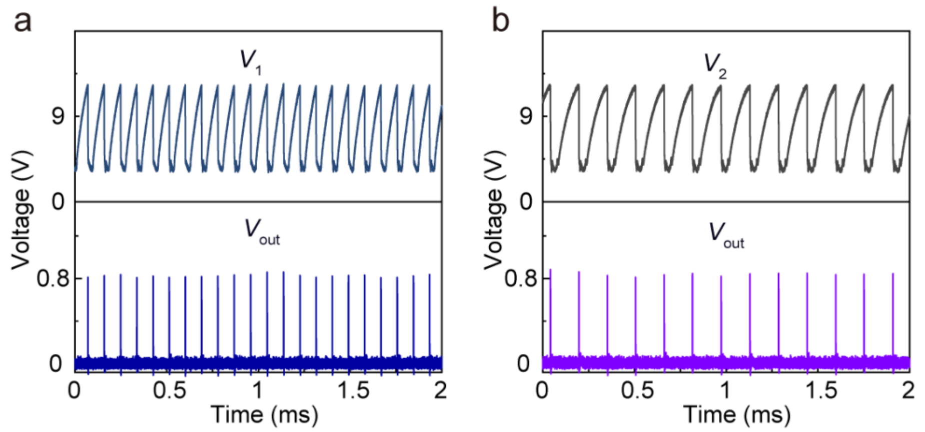


**Supplementary Figure 19. The output results of oscillator under different applied current. (a** and **b)** Experiment results of oscillator under only one current bias applied with 1.5 mA (a) and 1.22 mA (b), respectively.

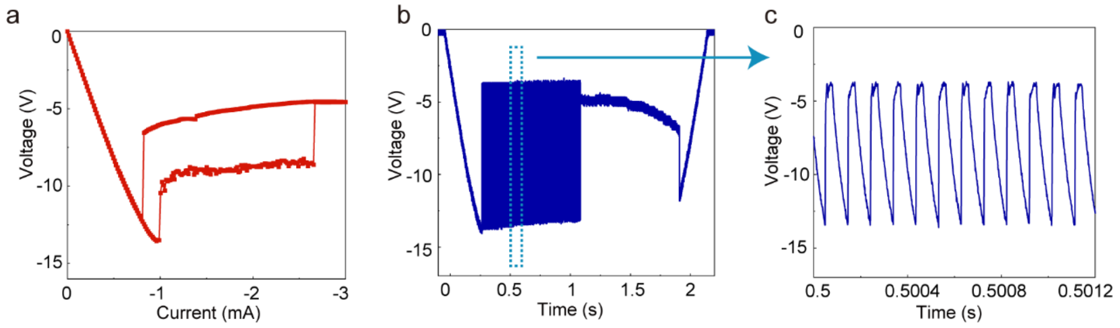


**Supplementary Figure 20. Quasi-static *I*-*V* characteristics of Au/$VO_2$/Au memristor. (a)** Current-driven current-Voltage (*I*-*V*) characteristics of the memristor. **(b)** Current-driven *I*-*V* characteristics of the device comes from the digital storage oscilloscope. **(c)** Zoom-in view of the green box.

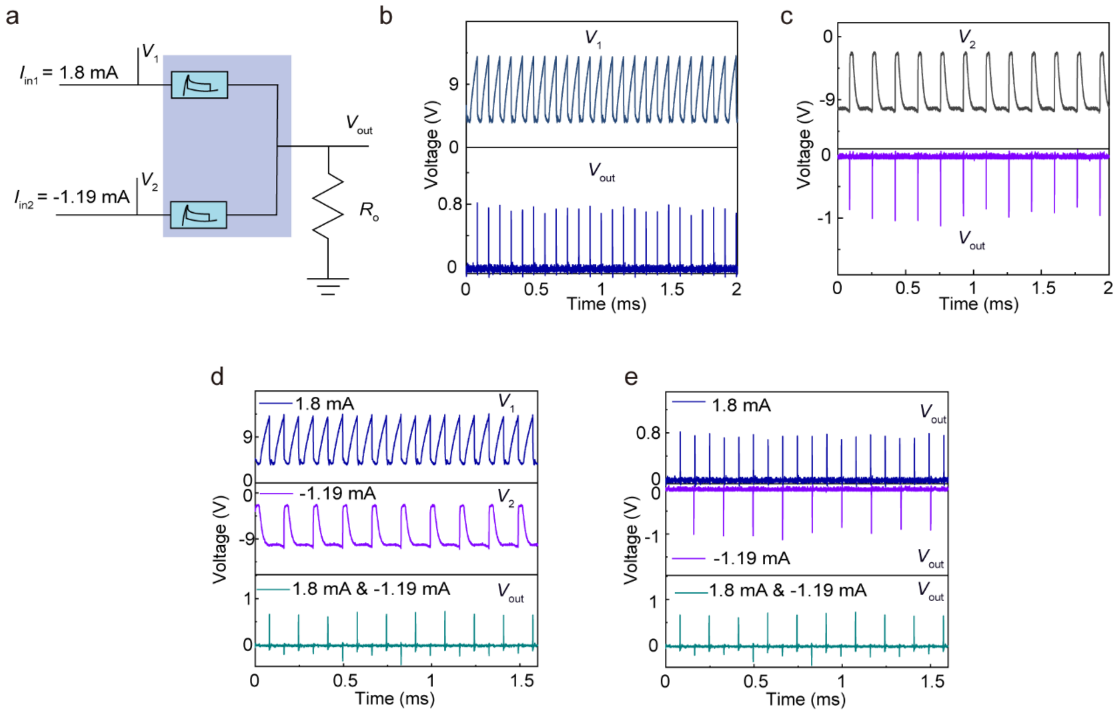


**Supplementary Figure 21. Frequency converter design using $VO_2$ memristor array-based oscillators. (a)** Schematic diagram of the integration of pulse signals accumulated through two oscillators. **(b-c)** Experiment results of oscillator under only one current bias applied with 1.8 mA (b) and -1.19 mA (c), respectively. **(d)** The output results with two current biases (1.8 mA and -1.19 mA) applied simultaneously. **(e)** The response $V_{out}$ with only one current bias 1.8 mA (first panel) or -1.19 mA (second panel) applied. The response $V_{out}$ with current biases applied simultaneously (third panel).

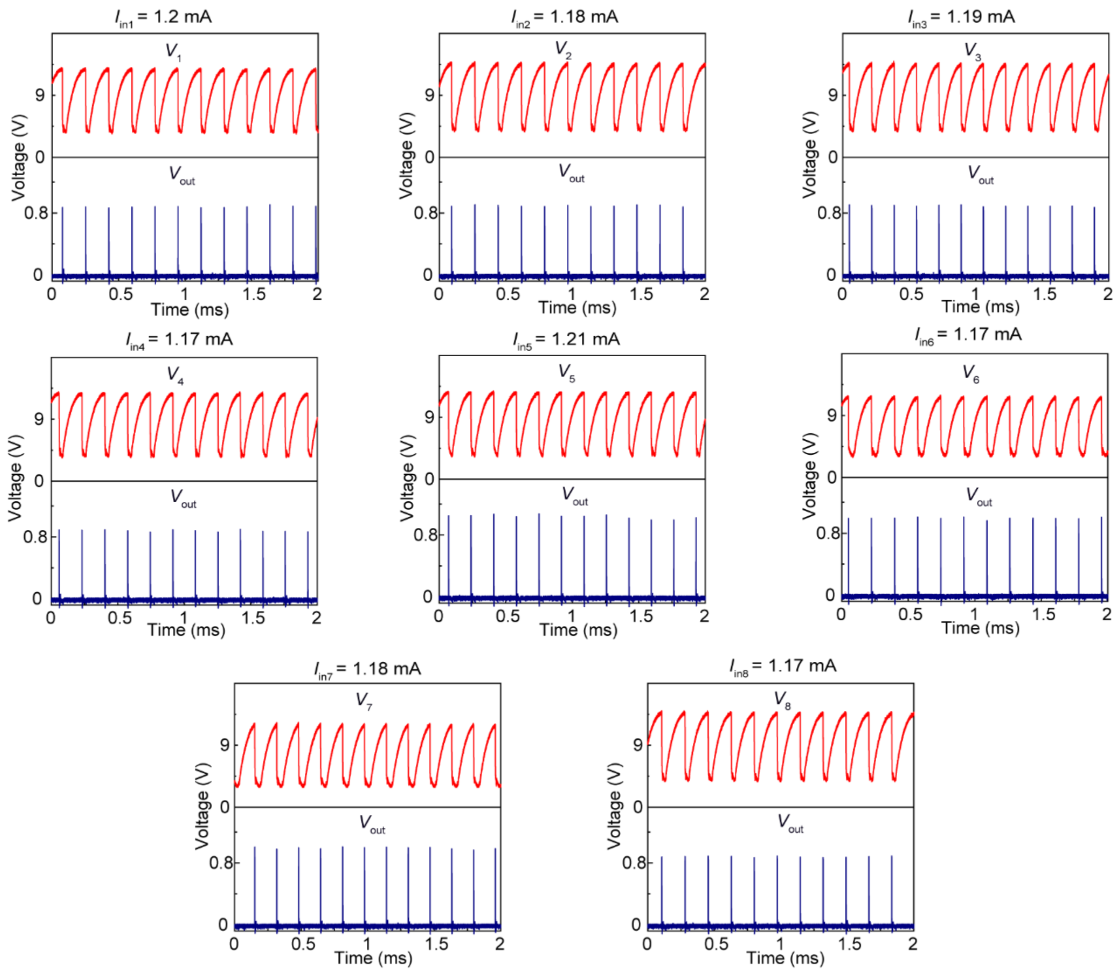


**Supplementary Figure 22. The output results of oscillator under different applied current.** Experiment results of oscillator under only one current bias applied, respectively.

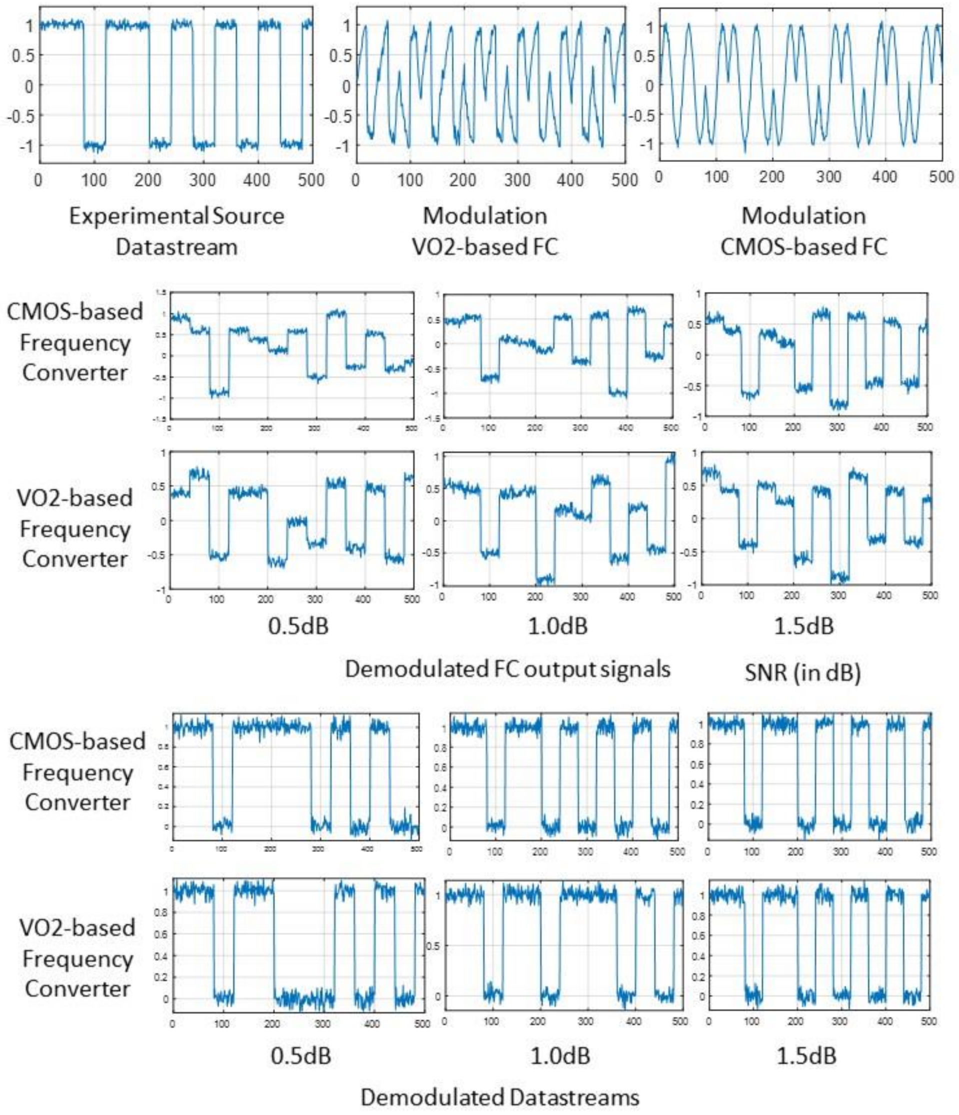


**Supplementary Figure 23.** Measurements of frequency synthesize and mix using CMOS-based frequency converter versus $VO_2$ memristor-based frequency converter on an experimental source datastreams from WIoT sensors. In this example, $VO_2$ memristor-based frequency converter reaches the same performance of CMOS-based frequency converter at SNR 1.5 dB.

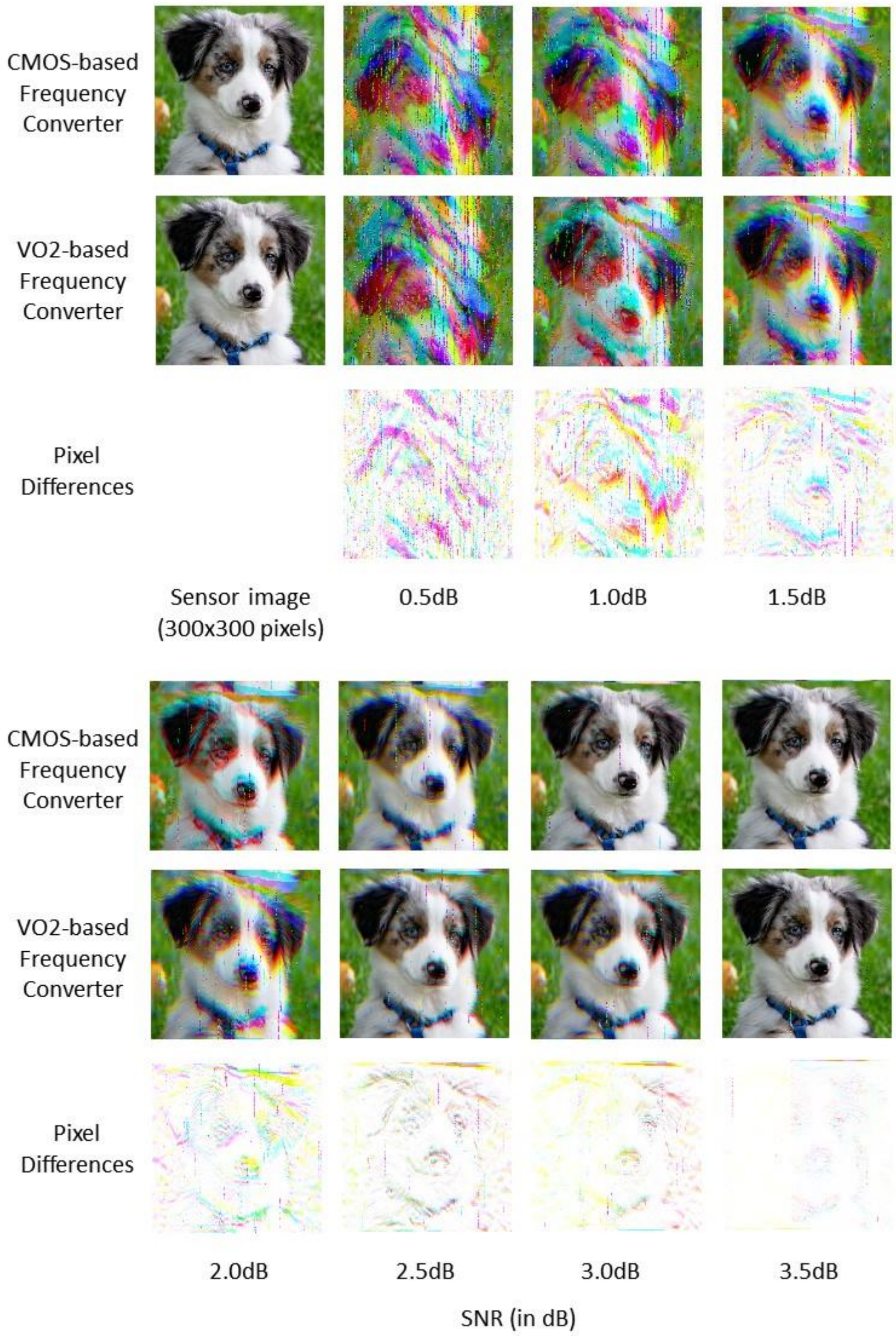


**Supplementary Figure 24.** Comparison of CMOS-based frequency converter versus $VO_2$ memristor-based frequency converter across different SNRs in vision WIoT.

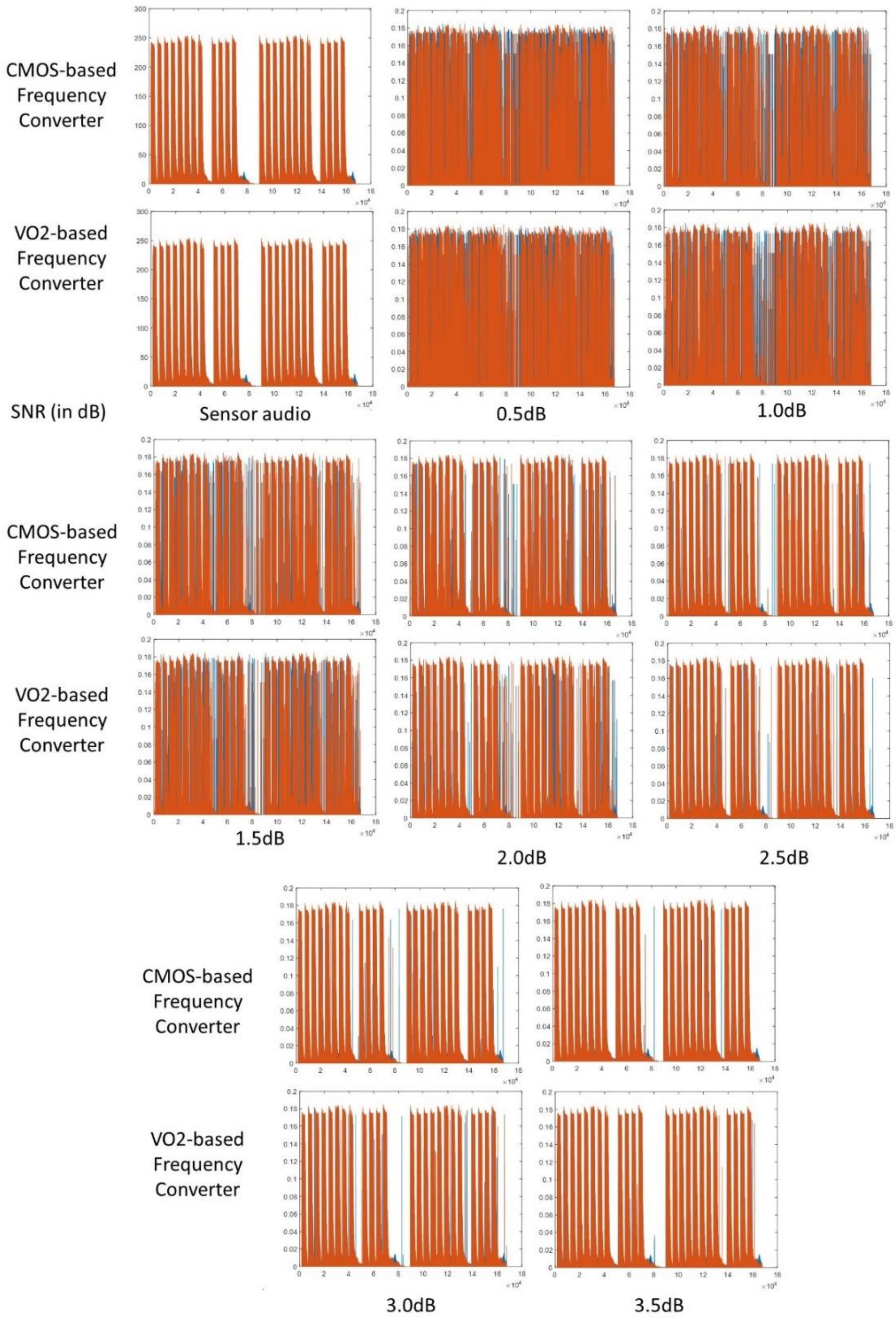


**Supplementary Figure 25.** Comparison of CMOS-based frequency converter versus $VO_2$ memristor-based frequency converter across different SNRs in audio WIoT.

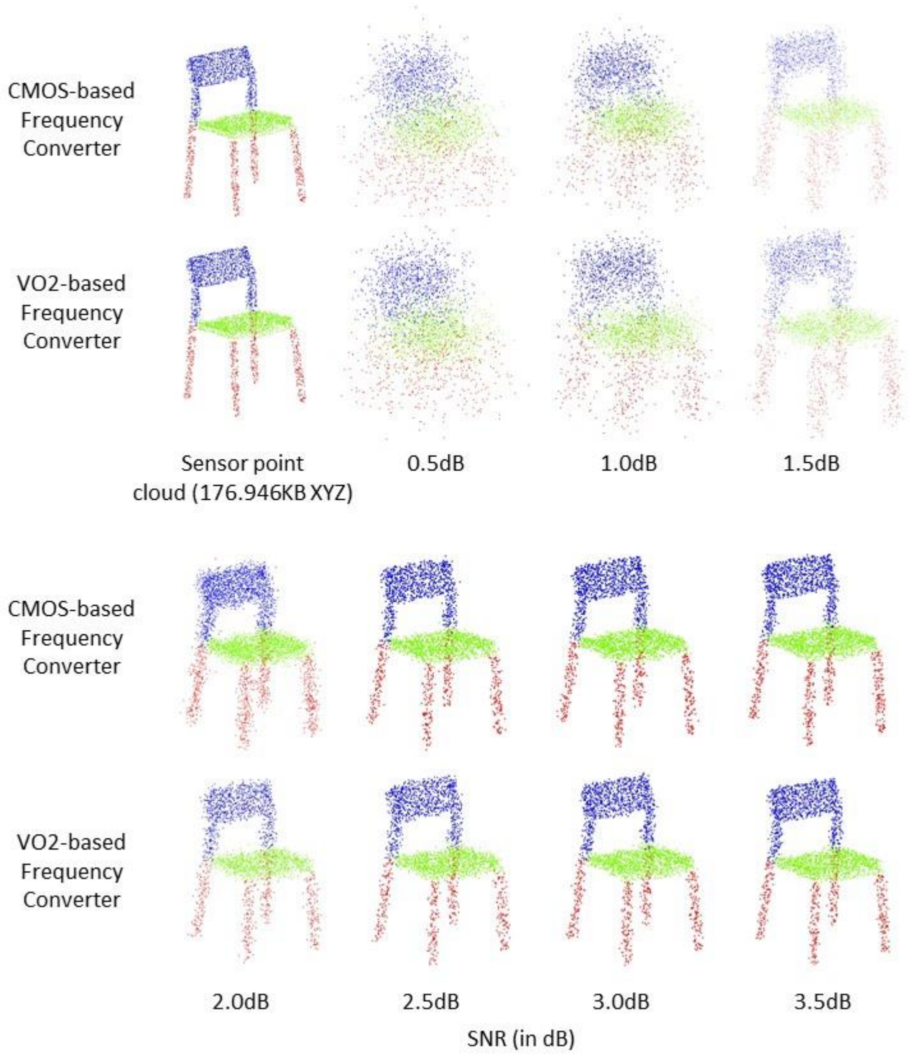


**Supplementary Figure 26.** Comparison of CMOS-based frequency converter versus $VO_2$ memristor-based frequency converter across different SNRs in point cloud WIoT.

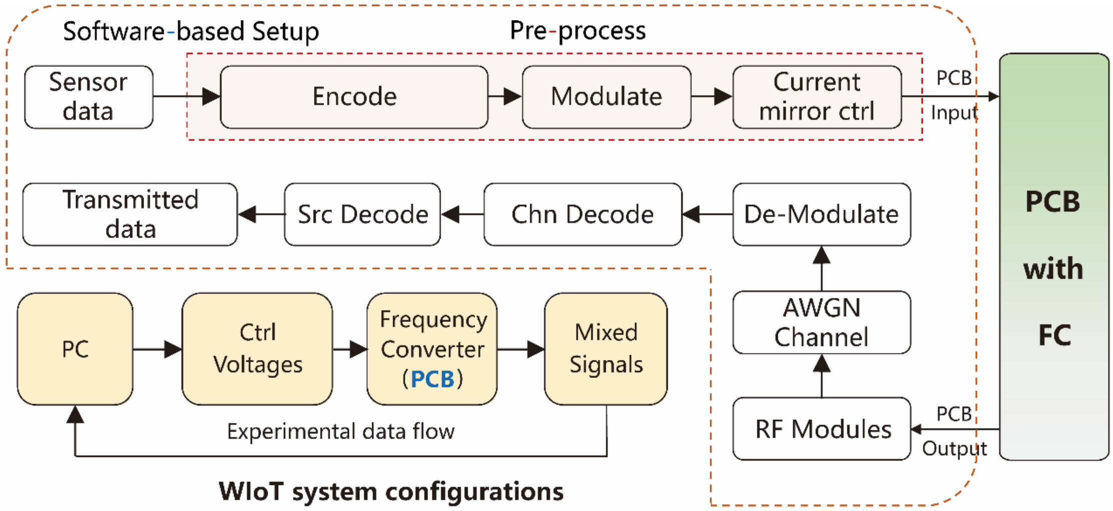


**Supplementary Figure 27.** Detailed illustration of each module in end-to-end wireless IoT demonstration system and interface between PCB and software.

Step ①: Sensor data are: 1) audio datastream of 3 seconds with sampling frequency of 4.41 kHz (each sampling point is quantized using FP32 and the total data size is 52.92 kB); 2) image datastream of 300p×300p (each pixel is quantized as INT8 RGB and the total data size is 270 kB); 3) point cloud datastream (with data size 176.946 kB). These data are sent to Encode block as a 1-dimensional bitstream and its length is determined by the sensor data size.

Step ②: The Encode block includes: 1) Huffman encoding given a vector of probability of the unique symbols. The probability is computed based on symbol count divided by the total length of pre-transmitted datastream. We use the built-in function huffmanenco in MATLAB for this process; 2) Convolutional coding for channel encoding, which uses a generator matrix of [1 1 1; 1 0 1] with code rate 1/2. The block diagram of the convolutional encoder is shown in Supplementary Figure 28. Depending on the number of frequency channels, the resulting datastream from Encode block can

remain as two 1-dimensional encoded bitstreams or demultiplexed into 8 1-dimensional encoded bitstreams.

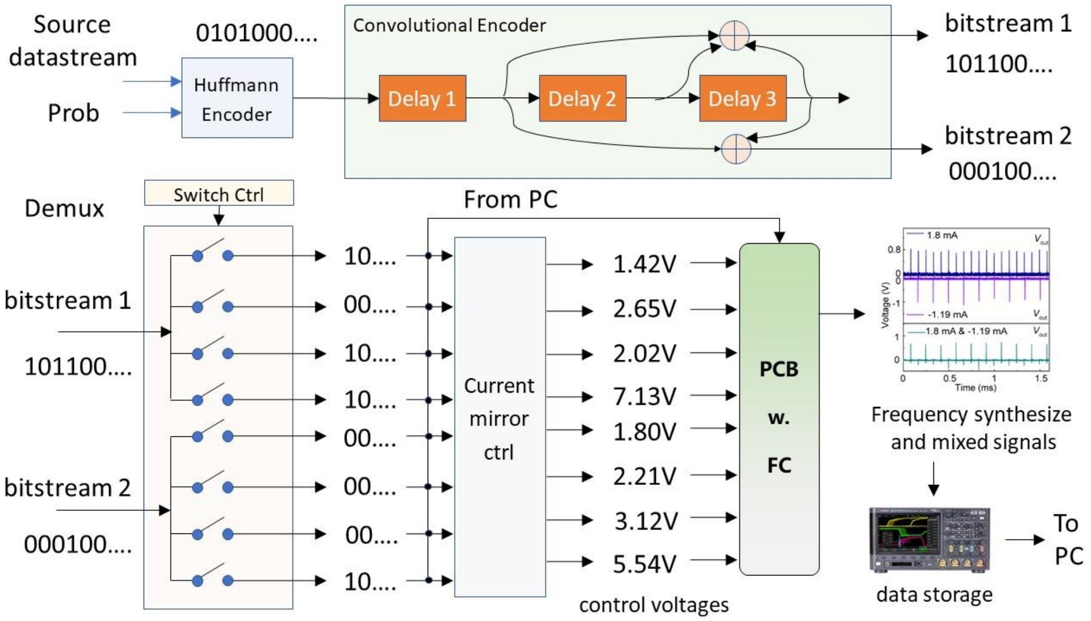


**Supplementary Figure 28.** Detailed illustration of encoding/modulate blocks and example interface signals with PC.

Step ③: The Modulate block receives the 2~8 1-dimensional bitstreams from Encode block and uses binary phase shift keying (BPSK) to map the input bit 0's or 1's. The 0's and 1's in the bitstreams are mapped to -1 and +1. These -1's and +1's data waves are sent to PCB and the current mirror ctrl block generates physical voltages controlling $VO_2$ drivers for in-situ frequency synthesize and mixing purpose. Note that the Current mirror ctrl block is still in software which generates current mirror ctrl voltages based on Supplementary Figure 14, 15, 16 and 22.

Step ④: The generated ctrl voltages are used to configure parallel voltage-controlled current sources from 0~2mA to drive the $VO_2$ memristors for follow-up frequency synthesize and mix on PCB.

Step ⑤: We use a delay-line based phase matcher to first manually adjust the phases of the $VO_2$ generated oscillation waves on PCB. Upon completion of the phase matching, we drive the $VO_2$ based FC using current sources and the in-situ synthesized and mixed signals are stored with oscilloscopes.

Step ⑥: The mixed analog signals are sent back to the software where the filter, attenuator, power amplifier and antenna modules are employed to simulate the data transmission through electromagnetic waves. These modules are written as MATLAB functions.

Step ⑦: The AWGN block adds white Gaussian noise to the radio frequency signals based on experimental SNR levels. The addition uses MATLAB awgn function.

Step ⑧: The Demodulate block receives noisy analog streams from AWGN block and map -1's and +1's back to 0's and 1's, respectively.

Step ⑨: The channel decoder block decodes the received bitstream using Viterbi decoder. The Viterbi decoder trellis is pre-determined by generator matrix G in step 2 and we use MATLAB vitdec function for this purpose.

Step ⑩: The src decoder block receives the channel decoded bitstream and decode the source bitstream based on Huffman coding scheme.

Supplementary Table 1. parameter of the device

| Parameter | Value | Unit |
|---|---|---|
| $\alpha$ | $5\times10^{-7}$ | $m^{-1}$ |
| $\rho_{0,ins}$ | $4.6\times10^{-7}$ | $\Omega$ m |
| $\rho_{0,met}$ | $4.8\times10^{-6}$ | $\Omega$ m |
| $E_{a,ins}$ | 0.2 | eV |
| $E_{a,met}$ | 0.06 | eV |
| $A$ | $3.4\times10^{-76}$ | - |
| $W$ | 5 | eV |
| $C_{th}$ | $5\times10^{-11}$ | J $K^{-1}$ |
| $R_{th}$ | $3.3\times10^{3}$ | K $W^{-1}$ |
| $T_{amb}$ | 298 | K |
| $R_s$ | 25 | k$\Omega$ |

Supplementary Table 2. Area Cost Estimation of $VO_2$-based frequency converter using 180 nm process technology.

| Circuit Module (180nm) | Number | Area ($mm^2$) |
|---|---|---|
| $VO_2$ Driver | 8 | 0.11 |
| $VO_2$ Memristor Array | 8×8 | 0.16 |
| Delay Line | 8 | 0.038 |
| DeMux | 2 | 0.013 |
| Periphery Circuits (Cap/Res/…) | 8 | 1.62 |
| Total | N/A | 1.941 |

Supplementary Table 3. Comparison with CMOS-based frequency converter

| Frequency Converter | | CMOS-based[1] (Qorvo) | CMOS-based[6] (Analog Devices) | $VO_2$ Memristor-based (This work) |
|---|---|---|---|---|
| Technology | | 40nm | 65nm | 180nm |
| Main Applications | | Radio-freq./WIoT | Radio-freq./WIoT | Energy-efficient WIoT |
| Frequency Range | | 30-2500MHz | 1KHz-3000MHz | Up to 48KHz |
| Latency | | > 5ns | $3\mu s$ | ~50ns |
| Area Cost | | 5mm×5mm (QFN) | 4mm×4mm (QFN) | $1.941mm^2$ |
| Synthesize & Mix | | Both | Mix Only | Both |
| Perf. (Acoustic/Vision/Spatial) | BER | 3.47dB/3.71dB /4.03dB | 3.47dB/3.71dB /4.03dB | 3.49dB/3.88dB /4.24dB |
| | Power | 165~225mW | 126~147mW | 85.2~114.1mW |

The reference CMOS-based frequency converters for comparison are implemented using more advanced process technology (from Qorvo[1] and Analog Devices[6]).

Supplementary Note 1:

We constructed a SPICE-based lumped-element compact model similar to those reported in ref. 2 for the $VO_2$ memristor used in this study. The $VO_2$ memristor is modelled as a highly-nonlinear temperature-dependent resistor with resistance given by Eq. 1[3]:

$$R_{\mathrm{VO2}}(T) = \frac{R_{\mathrm{ins}} \cdot R_{\mathrm{met}}}{f R_{\mathrm{ins}} + (1-f) R_{\mathrm{met}}} \tag{1}$$

$R_{\mathrm{ins}}$ and $R_{\mathrm{met}}$ are the resistance of the insulating phase and the metallic phase, respectively, while $f$ represents the volume fraction of the insulating phase (Eq. 2-4):

$$R_{\mathrm{ins}}(T) = \alpha \cdot \rho_{0,\mathrm{ins}} \exp\left(\frac{E_{\mathrm{a,ins}}}{k_{\mathrm{B}} T}\right) \tag{2}$$

$$R_{\mathrm{met}}(T) = \alpha \cdot \rho_{0,\mathrm{met}} \exp\left(\frac{E_{\mathrm{a,met}}}{k_{\mathrm{B}} T}\right) \tag{3}$$

$$f(T) = \frac{1}{1 + A \cdot \exp\left(\frac{W}{k_{\mathrm{B}} T}\right)} \tag{4}$$

$\rho_0$ is a resistivity constant, $E_a$ is the activation energy, $\alpha$ is a prefactor related to the device geometry, $A$ is a constant related to the temperature at which the insulator-metal transition takes place, while $W$ is another constant related to the width of transition.

The evolution of temperature with time follows Newton's law of cooling (Eq. 5):

$$C_{\mathrm{th}} \frac{dT}{dt} = -\frac{T - T_{\mathrm{amb}}}{R_{\mathrm{th}}} + I^2 R_{\mathrm{VO2}} \tag{5}$$

and is determined using a thermal circuit as shown in Supplementary Fig. 4. $T_{\mathrm{amb}}$ is the ambient temperature and $I$ is the current through the $VO_2$ memristor. The thermal

capacitance ($C_{th}$) is constant while the thermal resistance is a nonlinear function of temperature given by Eq. 6:

$$R_{\mathrm{th}}(T) = R_{\mathrm{th0}} + \beta f(1 - f) \tag{6}$$

which results in a spike in the thermal resistance near the transition, similar to that reported in ref. 4. $R_{th0}$ is the thermal resistance at temperatures far from the transition and $\beta$ is a constant. The values of all parameters are listed in Supplementary Table 1.

During the measurement of the *I*-*V* curve, the circuit oscillates due to parasitic capacitances when it is biased within the NDR region (~1 mA to 2.7 mA, Fig. 1d of the main text). However, the apparent steady-state characteristics can be attributed to the averaging of the voltage oscillations by the source measurement unit[5]. Hence, the *I*-*V* curve should be fitted using the averaging technique and a measurement circuit illustrated in Supplementary Fig. 4. Each voltage evolution under a constant current bias is averaged over time, resulting in individual data points used to plot the simulated curve in Fig. 1d of the main text.

## Supplementary References


1. Fractional-N Synthesizer with Integrated Frequency Mixers, Qorvo, Inc. https://www.qorvo.com/products/frequency-converters/integrated-synthesizers-with-mixers

2. Gibson, G. A. et al. An accurate locally active memristor model for S-type negative differential resistance in $NbO_x$. *Appl. Phys. Lett.* **108**, 023505 (2016).

3. Zhong, X. et al. Avalanche breakdown in microscale $VO_2$ structures. *J. Appl. Phys.* **110**, 084516 (2011).

4. Brown, T. D. et al. Electro-thermal Characterization of Dynamical $VO_2$ Memristors via Local Activity Modeling. *Adv. Mater.* 2205451 (2022).

5. Das, S. K. et al. Physical Origin of Negative Differential Resistance in $V_3O_5$ and Its Application as a Solid-state Oscillator. *Adv. Mater.* 2208477 (2022).

6. 1kHz-3000MHz High Signal Level Active Mixer, Analog Devices, Inc. https://www.analog.com/en/products/lt5512.html.
