# Supplementary material for: VO2 memristor-based frequency converter with in-situ synthesize and mix for wireless internet-of-things
Source: Nat Commun. 2024 Feb 19;15:1523. doi: 10.1038/s41467-024-45923-7 (PMC10876666; doi:10.1038/s41467-024-45923-7)
Supplement: Supplementary file 3 — Description of Additional Supplementary Files [file 41467_2024_45923_MOESM3_ESM.pdf]

Description of Additional Supplementary Files

Supplementary Movie 1. Movie showing filament formation in planar VO2 devices. The lower panel is the optical microscope video, meanwhile, the upper panel is the generated spiking signal V_{mem}.
